# First complete chloroplast genome of the rare medicinal plant *Schnabelia tetrodonta*

**DOI:** 10.1080/23802359.2021.1975507

**Published:** 2021-09-20

**Authors:** Fengming Ren, Liqiang Wang, Wei Zhuo, Shenge Lu, Xiaofu Zhu, Chao Liu, Meisen Yang

**Affiliations:** a Research and Utilization on Characteristic Biological Resources of Sichuan and Chongqing Co-construction Lab, Chongqing Institute of Medicinal Plant Cultivation, Chongqing, China; b College of Pharmacy, Heze University, Heze, China; c Key Laboratory of Novel Food Resources Processing, Ministry of Agriculture and Rural Affffairs/Institute of Agro-Food Science and Technology, Shandong Academy of Agricultural Sciences, Jinan, China; d Chinese Herbal Medicine Industry Center of Xiushan Tujia and Miao Autonomous County, Chongqing, China

**Keywords:** Chloroplast genome, *Schnabelia tetrodonta*, medicinal plant, phylogenetic analysis, Lamiaceae

## Abstract

*Schnabelia tetrodonta* is a medicinal plant used in traditional Chinese medicine. However, the molecular biology data of the species was too scarce to bioprospect the medicinal species. In this study, the first complete chloroplast genome (cp) of *S. tetrodonta* was sequenced and assembled based on the next generation sequencing. The cp genome is 157,004 bp in length, including a large single-copy (LSC) region of 83,605 bp, a small single-copy (SSC) region of 36,899 bp, and a pair of inverted repeat (IR) regions of 18,250 bp each. The genome encodes 134 genes, including 90 protein-coding genes, 36 tRNA genes, and 8 rRNA genes. The GC content of whole genome is 37.80%. The phylogenetic analysis based on 20 complete cp sequences (19 genome sequences from the Teucrioideae of Lamiaceae and an outgroup of *Ipomoea purpurea*) revealed that *S. tetrodonta* was closely related to *S. oligophylla.*

*Schnabelia tetrodonta* (Sun) C. Y. Wu et C. Chen is a member of the genus *Schnabelia* in Lamiaceae. It is an Endangered species, narrowly distributed in the mountainous areas of central Sichuan province and northern Guizhou province of China (Li and Hedge 1994). *Schnabelia tetrodonta* is rich in flavonoids, polysaccharides and saponins (Dou et al. 2002, 2003; Zhou et al. 2004). It is used as a traditional Chinese medicine to treat rheumatic joint pain. Modern pharmacological studies show that *S. tetrodonta* has anti-fatigue, anti-oxidant, anti-inflammatory, analgesic and immunomodulatory effects (Ouyan). However, S*. tetrodonta,* as an important medicinal plant, has no genome information reported up to now. In this study, the first chloroplast (cp) genome of the species was sequenced for excavating and protecting the resource of *S. tetrodonta*.

Fresh leaves of *S. tetrodonta* were collected from Nanchuan, Chongqing, China (107°21′ E, 29°13′ N, 591 m). The voucher specimen was conserved in Chongqing Institute of Medicinal Plant Cultivation under the accession number of CIMPC-RFM-20210301 (Contact person: Fengming Ren; Email: 348080877@qq.com). A modified CTAB-based method was used to extract the whole genomic DNA, and the purity and integrity of the DNA were analyzed by Nanodrop (Thermo Fisher Scientific) and agarose gel electrophoresis. Total DNA was used to generate libraries with insert size of 350 bp and was generated 3.43 Gb raw reads by Illumina Hiseq 2500 Platform (Illumina, Hayward, CA, USA). Low-quality readings and adapters in the raw data were deleted by trimmomati (version 0.35) with default paprameters (Bolger et al. 2014). Using the clean data with 150 bp paired-end read lengths obtained from the raw data, a cpgenome was assembled by NOVOPlasty (version 4.1) with the default parameters (Nicolas et al. 2016) and annotated by CPGAVAS2 (Shi et al. 2019). After manual check and adjustment, the annotated cp genome was submitted to GenBank (MW928532). The complete cp genome of *S. tetrodonta* was 157,004 bp in size and exhibited a typical angiosperm circular cp structure, containing four regions: large single-copy region (LSC: 96,530 bp), small single-copy region (SSC: 9,636 bp), and a pair of inverted repeats (IR: 41,955 bp). The GC content was 37.80% (whole genome), 36.16% (LSC), 37.72% (SSC) and 41.64% (SSC). The GC content of the genome and each genomic region was also typical of angiosperm cp structure. The genome encoded 134 genes, including 90 protein-coding genes, 36 tRNA genes, and 8 rRNA genes.

Nineteen genome sequences from the Teucrioideae of Lamiaceae were downloaded from the NCBI database. The genome sequence of *Ipomoea purpurea* Lam. was used as an outgroup. Finally, a total of twenty cp genome sequences were multi-aligned by MAFFT software (version 7.487) with the parameter of ‘–auto’ (Katoh and Standley 2013). Based on the aligned sequences, a Maximum-likelihood phylogenetic tree was built by IQ-TREE (version 1.6.12) (Lam-Tung et al. 2015) with 1000 bootstrap replicates under parameters of ‘-nt AUTO -m MFP -bb 1000 -bnni’. Phylogenetic analysis showed that *S. tetrodonta* was closely related to *S. oligophylla* (Figure 1). *S*chnabelia *oligophylla* is the only other species of *Schnabelia* with available plastome data. So the result is expected, which also support the reliability of our data. However, the reliability of the phylogenetic state of *S. tetrodonta* was not high because of scarce available *Schnabelia* plastome data. To eliminate the phylogenetic state of *Schnabelia*, more available *Schnabelia* plastome data should be produced in the further studies. In this study, to sequence the plastome of *S. tetrodonta* is a step forward in tackling this lack of information.

**Figure 1.**
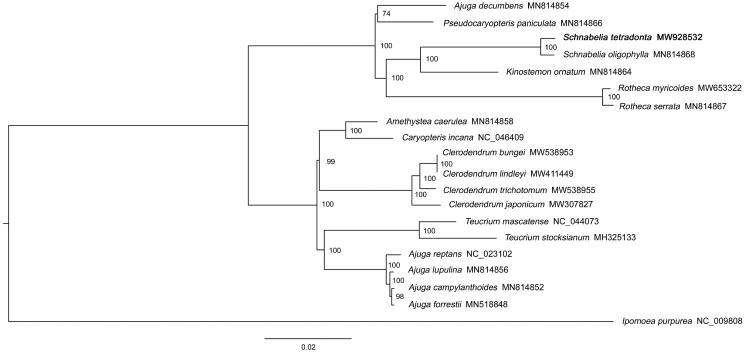
Maximum likelihood phylogenetic tree based on the chloroplast genome sequences of 19 Teucrioideae (Lamiaceae) species and *Ipomoea purpurea* (outgroup). The GenBank accession numbers is behind the Latin name. The bootstrap support values are beyond each node in the tree *S. tetrodonta* is marked by bold font.

## Data Availability

The genome sequence data that support the findings of this study are openly available in GenBank of NCBI at https://www.ncbi.nlm.nih.gov, under the accession No. MW928532. The associated Bio-Project, Bio-Sample and SRA numbers are PRJNA543381, SAMN19416297, and SRR14688233, respectively.
